# Complex-Forming Properties of Ceftazidime with Fe(III) Ions in an Aqueous Solution

**DOI:** 10.3390/molecules27217226

**Published:** 2022-10-25

**Authors:** Marek Pająk, Magdalena Woźniczka, Jakub Fichna

**Affiliations:** 1 Department of Physical and Biocoordination Chemistry, Medical University of Lodz, Muszyńskiego 1, 90-151 Lodz, Poland; 2 Department of Biochemistry, Medical University of Lodz, Mazowiecka 5, 92-215 Lodz, Poland

**Keywords:** Fe(III) complexes, ceftazidime, UV-vis spectroscopy, potentiometry

## Abstract

In the present study, the complexing properties of ceftazidime with Fe(III) ions in aqueous solutions were characterized by UV-vis spectrophotometric and potentiometric methods. Using the UV-vis spectrophotometric method, the absorbance values for Fe(III) ions, a third-generation cephalosporin antibiotic (ceftazidime), and the Fe(III)-ceftazidime system were determined. Based on pH-metric studies, the value of the stability constant for the Fe(III)-ceftazidime complex was calculated.

## 1. Introduction

Iron(III) ion is the fourth most important transition metal ion in biological systems [1]. It is an essential micronutrient for humans, with critical functions in many cellular processes, including DNA synthesis, replication, repair, and transcription [2]. Iron functions as a redox catalyst and occurs as ferrous Fe^2+^ or ferric Fe^3+^ inside the cell [3,4]. The deficiency of iron decreases oxygen delivery to cells, resulting in fatigue, poor work performance, and decreased immune function [5]. Excess amounts of Fe(III) ions in a living cell can catalyse the production of reactive oxygen species through the Fenton reaction, which can damage lipids, nucleic acids, and proteins. The cellular toxicity of Fe(III) contributes to serious diseases, like Huntington’s, Alzheimer’s, and Parkinson’s diseases [6].

**Figure 1:**
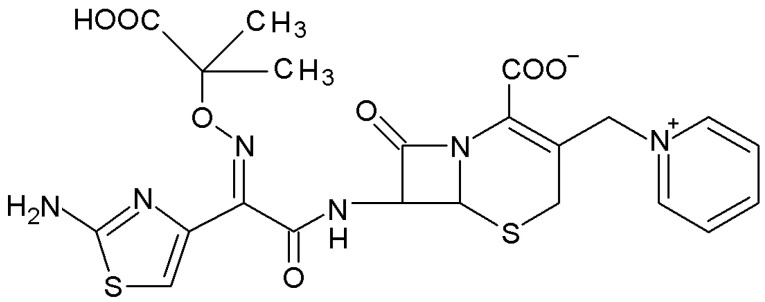
Structure of ceftazidime.

Ceftazidime (Figure 1) is a third-generation cephalosporin antibiotic usually reserved for the treatment of infections caused by *Pseudomonas aeruginosa*. It is also used in combination with other antibiotics for the empirical treatment of febrile neutropenia [7,8]. In general, cephalosporins are usually bactericidal against susceptible bacteria and act by inhibiting mucopeptide synthesis in the cell wall, resulting in a defective barrier and an osmotically unstable spheroplast [9]. The mechanism for this effect has not been definitively determined, but beta-lactam antibiotics have been shown to bind to several enzymes (carboxypeptidases, transpeptidases, endopeptidases) within the bacterial cytoplasmic membrane that is involved in the cell wall synthesis [10].

Many cephalosporin antibiotics form stable complexes with *d*-metal cations. Studying the metal complexes of antibiotics is of interest for both the design of new combined drugs and the development of new procedures of drug analysis based on complexation reactions. By the present time, solid M(Ctzd)Cl ceftazidime complexes, where M = Mn(II), Fe(II), Co(II), Ni(II), Cu(II), or Cd(II), have been obtained as precipitates from methanol solutions. Elemental analyses agree with a 1:1 metal to ligand stoichiometry for all the complexes. The manganese(II) and cobalt(II) complexes are beige and dark red, respectively, while the iron(II), nickel(II), and copper(II) complexes are green. The cadmium(II) complex is white, and the complexes are air-stable solids that are insoluble in H_2_O and other common organic solvents such as EtOH, benzene, acetone, acetonitrile, ether, DMF, and DMSO. The general formula [M(ceftaz)(H_2_O)Cl] has been assigned to the complexes. The insolubility and high melting points of the complexes (4300 °C) suggest that they are polymeric. Thermograms of the hydrated metal complexes indicate endothermic decompositions in the 150–160 °C range due to the loss of coordinated water and also reveal that the complexes are stable and have no hydration water or solvent [11]. The synthesis and spectrophotometric and electrochemical characterization of the complexation of Schiff base (ceftazidime “CFZ”-p-dimethylaminobenzaldehyde “DAB”) with Cu(II), Co(II), Ni(II), Fe(III), and Ru(III) ions is also described. The obtained precipitates were filtered, washed with methanol, and dried in the air. The analytical data show the composition of the metal complex to be [M(CFZ-DAB)Cln]Cl, where CFZ-DAB is the Schiff base ligand; n = 1 for the Cu(II), Co(II), and Ni(II) complexes; and n = 2 for the Fe(III) and Ru(III) complexes. The conductance data indicate that all the complexes are strong electrolytes. The compound (CFZ-DAB) behaves as a tridentate ligand. However, the obtained complexes have a mononuclear nature. The electrochemical properties of the metal complexes were investigated by cyclic voltammetry (CV) using glassy carbon electrode. The oxidation/reduction of metal complexes was irreversible/reversible and exhibited a diffusion-controlled process depending on the pH. The dependence of intensities of currents and potentials on the pH, concentration, scan rate, and nature of the buffer was investigated [12].

This work aimed to evaluate the complexation properties of a cephalosporin antibiotic (ceftazidime) with Fe(III) ions in an aqueous solution. The absorbance values for Fe(III) ions, ceftazidime, and the Fe(III)-ceftazidime system were determined by the UV-vis spectrophotometric method. The value of the stability constant for the Fe(III)-ceftazidime system was calculated using the potentiometric method.

## 2. Results and Discussion

**Figure 2:**
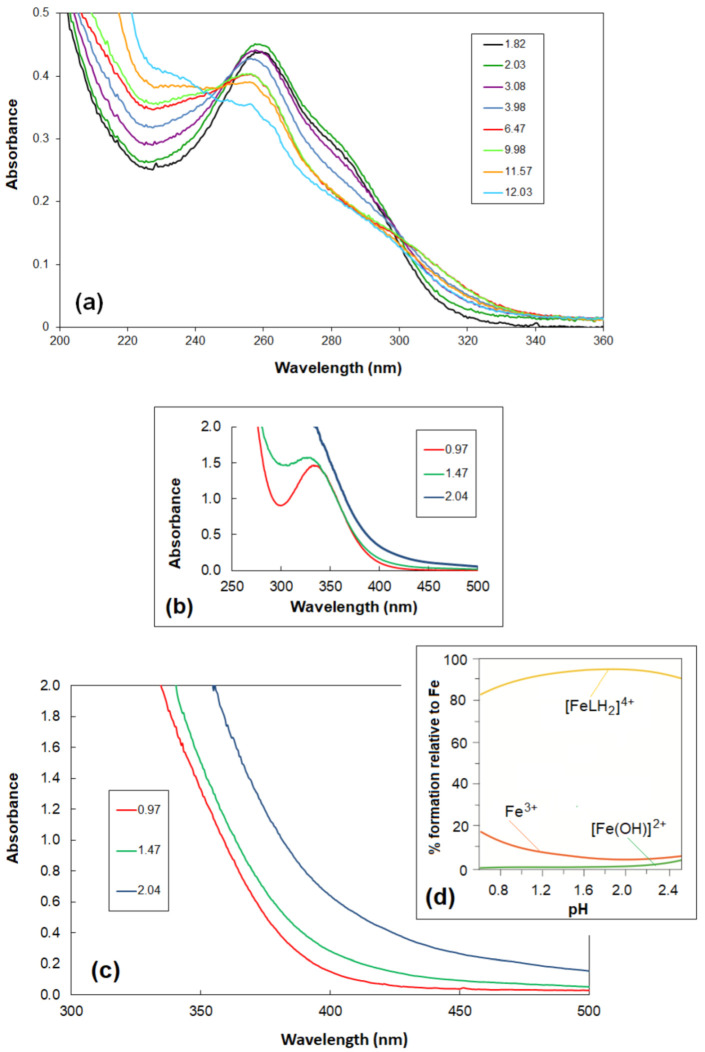
(**a**) UV spectra of ceftazidime within the pH range of 1.82–12.03, C_ceftazidime_ = 1.0·10^−5^ M; (**b**) UV-vis spectra of Fe(III) ions within the pH range of 0.97–2.04, $C_{{\mathrm{FeCl}}_{3}\cdot {6H}_{2}O}$ = 1.8∙10^−3^ M; (**c**) UV-vis absorption spectra of the Fe(III)-ceftazidime system, within the pH range of 0.97–2.04; $C_{{\mathrm{FeCl}}_{3}\cdot {6H}_{2}O}$ = 1.8·10^−3^ M, C_ceftazidime_ = 3.6·10^−3^ M; (**d**) The distribution diagram of species as a function of pH for the complex formed in the Fe(III)-ceftazidime system at a ligand–Fe(III) molar ratio of 5:1, C_ceftazidime_ = 3.6·10^−3^ M obtained from potentiometric data.

The electronic absorption spectra for ceftazidime water solution in the range of 200–360 nm revealed absorbance features at ca. 235^sh^, 257, 283^sh^, and 300^sh^ nm within the pH range of 1.82–12.03 (Figure 2a). The presence of isosbestic points (at ca. 235, 245, 250, and 300 nm) confirms the existence of equilibria between the various deprotonated forms of ceftazidime [13,14].

The electronic absorption spectra for the Fe(III) chloride water solution in the range of 250–500 nm revealed absorbance features at ca. 337 nm at the pH 0.97 (Figure 2b). The hydrolysis of the Fe(III) ions was already observed above pH 1.0 and corresponded to the literature data. As indicated by the reference data, the formation constants of the aqua-hydroxido complexes [Fe(OH)]^2+^ and [Fe(OH)_2_]^+^ are equal to log *β*_10-1_ = −2.68 and log *β*_10-2_ = −6.48, respectively [15,16,17,18,19].

Figure 2c shows the course of spectrophotometric titration in the range of pH = 0.97–2.04 at wavelengths of 300–500 nm. The Fe(III)-ceftazidime complexes are formed in a very acidic medium (at pH < 1.0) just at the beginning of titration. The absence of an absorption band at about 337 nm, at the pH 0.97—characteristic of free Fe(III) ions (Figure 2d)—is evidence of the formation of complexes. Above pH 2.0, a precipitate appears.

Calculations based on potentiometric titrations in the presence of the metal ion at a pH of approximately 2.00 confirmed the formation of the [FeLH_2_]^4+^ complex in an aqueous solution. The value of the determined overall stability constant is log *β* _[FeLH_2_]^4+^_ = 11.88 {Fe^3+^+2H^+^+L^−^ = [FeLH_2_]^4+^}. The related stability constant is equal to $\log K_{{\left[{\mathrm{FeLH}}_{2}\right]}^{4+}}^{\mathrm{Fe}}=11.88-7.62=4.26$ based on the equation $\log K_{{\left[{\mathrm{FeLH}}_{2}\right]}^{4+}}^{\mathrm{Fe}}=\log\beta_{112}-\log\beta_{012}$. In the literature, the values of the stability constants for Ni(II) and Cu(II) ions with ceftazidime are log *β* _NiCtzd+_ = 4.04 and log *β* _Ni(Ctzd)2_ = 6.41, log *β* _CuCtzd+_ = 5.03, respectively [13]. In the Hyperquad model were used the dissociation constants of ceftazidime, previously determined by pH-metric titration (log *β* [LH_3_]^2+^ = 9.23, log *β* [LH_2_]^+^ = 7.62, log *β* [LH] = 4.82) [13], where [LH_3_]^2+^ non-deprotonated all active groups, viz., two carboxyl (at the six-membered dihydrothiazine ring and at the methyl-ethoxy group), amine, and amide groups; [LH_2_]^+^ deprotonated a carboxyl group at the six-membered dihydrothiazine ring; and [LH] deprotonated two carboxyl groups. The hydrolysis constants of Fe(III) (log *β* [Fe(OH)]^2+^ = −2.68; log *β* [Fe(OH)_2_]^+^ = −6.48) were taken from the data [15]. The ionic product of water pK_w_ included in the equilibrium model was 13.77 [20]. The additional protonated and deprotonated complexes introduced to the equilibrium model were rejected during the procedure refinement. Figure 2d shows a representative species distribution for the Fe(III)-ceftazidime system based on the potentiometric determination of both the overall protonation constants of ceftazidime [13] and the stability constant of the complex.

We read in the literature that similar [Fe_2_(ceftazidime)_3_Cl_2_(H_2_O)(OH)] complexes have been synthesized, but in a solid product. A solution of ceftazidime and Fe(III) was added to hot ethanol. The solution was refluxed, filtered, and dried, which led to the formation of a solid product [21]. In the work are given physical measurements and analytical data of the complexes (elemental analysis, colour). The conformational changes and binding of ceftazidime in response to transition metals were identified by IR, electronic spectra, ESR, and magnetic susceptibility. Also, thermal analysis of ceftazidime and its metal complexes was conducted based on thermo-gravimetric and differential analysis curves. The mechanism of decomposition and kinetic parameters were evaluated. From magnetic measurement and spectral data, octahedral structures were proposed for permanent Fe(III)-ceftazidime complexes. The authors of the article also stated that ceftazidime complexes show higher positive antibacterial activity compared to antifungal activity. Other studies indicate the detrimental effects of iron overload in the setting of viral infections: the viruses seem to prosper in the presence of unbound iron. Therefore, iron chelation appears to be a potential and logical beneficial adjuvant therapy for viral infections in an era of multidrug-resistant viruses [22]. The pathogens require iron as a nutrient; iron deprivation serves as an innate immune mechanism against invading pathogens [23]. On the bases of Overtone’s concept and chelation theory, most metal complexes have higher activity than free ligands [24]. Thus, it can also be assumed that the activity of Fe(III)-ceftazidime complexes in an aqueous solution will be higher than that of commercial ceftazidime. 

The UV-vis spectrophotometric and potentiometric methods confirmed the possibility of forming a mononuclear complex in a mixture of ceftazidime with Fe(III) ions in an acidic medium. The results of our research could be helpful in identifying active sites of biomolecules, determining metal–ligand coordination, and designing biochemical syntheses, drugs, and biomarkers in medicine.

## 3. Materials and Methods

### 3.1. Reagents

Ceftazidime ((6R,7R,Z)-7-(2-(2-aminothiazol-4-yl)-2-(2-carboxypropan-2-yloxyimino)acetamido)-8-oxo-3-(pyridinium-1-ylmethyl)-5-thia-1-aza-bicyclo[4.2.0]oct-2-ene-2-carboxylate) was obtained from Sigma-Aldrich (St. Louis, MO, USA). Carbonate-free 1.0 M NaOH solution was purchased from J.T. Baker (Radnor, PA, USA). Perchloric acid solution from Laborchemie Apolda (Apolda, Germany) was standardized by titrations with NaOH. A standard solution of sodium perchlorate monohydrate (Laborchemie Apolda, Germany) was used to adjust the ionic medium. Argon of high purity (Linde, Dublin, Ireland) was used. Iron(III) chloride was obtained from Sigma-Aldrich, and 1.0 M HCl solution was prepared from concentrated hydrochloric acid (Avantor Performance Materials, Gliwice, Poland). All solutions were prepared in double-distilled water.

### 3.2. Spectrophotometric Measurements

Electronic spectra under argon were recorded on a Cary 50 Bio spectrophotometer, equipped with a fibre-optic device (with a path length of 1 cm, 5 mm), (Varian Pty. Ltd., Mulgrave, Australia). This enabled the study of equilibrium systems spectrophotometrically, simultaneously with pH measurements controlled by a Titrando 905 automatic titration (Metrohm, Herisau, Appenzell Ausserrhoden, Switzerland) kit with a combined InLab Semi-Micro (Mettler Toledo, Columbus, OH, USA) polymer microelectrode. Due to the highly disturbing absorption of the nitrate ion at about 300 nm, all the UV experiments were carried out in a perchlorate medium, which was enabled by a combined polymer microelectrode. The ionic strength (*I* = 1.0 M) was adjusted with NaClO_4_. The electrode was calibrated with buffers at pH 4.00 and 7.00 before use. The fibre-optic probe, 5 mm long, corresponding to a path length of 1 cm, was dipped directly into the thermostated titration vessel (a constant temperature of 25.0 ± 0.1 °C was maintained). A stream of pure argon was passed over the sample surface to obtain oxygen and carbon dioxide solutions freely. After each addition of carbonate-free NaOH and an appropriate time delay to equilibrate the system, the pH and EMF were controlled. The spectrum was recorded with a slow scan (300 nm min^−1^) at selected pH values.

The tests were first performed for the metal in the absence of the ligand (the total concentration of FeCl_3_ ∙ 6H_2_O was equal to 1.8·10^−3^ mol·L^−1^). Next, the ligand was examined in the absence of the metal (the total concentration of ceftazidime was equal to 1.0·10^−5^ mol·L^−1^). The solutions containing Fe(III) ions and ceftazidime were prepared with a ligand–metal molar ratio of 2:1 for two samples of the solution (the total metal concentrations were 1.8·10^−3^ mol·L^−1^ and 3.6·10^−4^ mol·L^−1^, respectively), and with a ligand–metal molar ratio of 1:2 (the total metal concentration was 7.1·10^−4^ mol·L^−1^). UV-vis spectra were recorded in the range of 200–900 nm, in an aqueous solution, and ionic strength *I* = 1.0 M (NaClO_4_). The studies were carried out at 25.0 ± 0.1 °C in a closed thermostated vessel, in anaerobic conditions under argon. The titrations were performed with the carbonate-free 1.0 M NaOH.

### 3.3. pH-Metric Titrations

Potentiometric titrations were conducted by using an automatic titrator system, the Titrando 905 (Metrohm, Herisau, Appenzell Ausserrhoden, Switzerland). An LL Biotrode combined glass electrode (Metrohm, Herisau, Appenzell Ausserrhoden, Switzerland) was calibrated with NaOH regarding the hydrogen ion concentration [25]. The solution of the acid was calibrated alkalimetrically and determined by the Gran method [26,27]. The measurements were carried out in a thermostated vessel at a constant temperature of 25.0 ± 0.1 °C and an ionic strength of 0.5 M (KCl). All titrations were carried out in aqueous solutions in 4 mL samples. Pure argon was passed over the solution surface.

The system was tested at ceftazidime–Fe(III) molar ratios of 5:1 and 10:1 at a pH of approximately 2.00 (the total concentration of ceftazidime was equal to 3.6 × 10^−3^ M) (Figure S1). The fitting procedure using the Hyperquad 2013 software allowed the calculation of the concentration formation constants according to the formula: *β*_mlh_ = [M*m*L*_l_*H*_h_*]/[M]*^m^*[L]*^l^*[H]*^h^*. The goodness of fit was checked by the objective function *U = Σ_i=1,m_ W_i_ r_i_^2^*, where *W* is the weight, *r* is a residual (equal to the difference between observed and calculated EMF values), *m* is the number of experimental points, and *n* is the number of refined parameters. The weighting factor *W_i_* is defined as the reciprocal of the estimated variance of measurements, dependent on the estimated variances of EMF and volume readings. The value of the normalized sum of squared residuals, *δ = U/(m − n)*, was compared with the *χ*^2^ (chi-squared) test of randomness at a number of degrees of freedom equal to *m − n* [28]. The speciation diagrams were simulated via the HySS 2009 software [29].

## Data Availability

Not applicable.

## Supplementary Materials

The following supporting information can be downloaded at https://www.mdpi.com/article/10.3390/molecules27217226/s1: Figure S1: The pH-titration curves for the Fe(III)-ceftazidime system, with a ligand–Fe(III) molar ratio of 5:1, *C*_ceftazidim*e*_ *=* 3.6 × 10^−3^ M.
