# Supplementary material for: Complex-Forming Properties of Ceftazidime with Fe(III) Ions in an Aqueous Solution
Source: Molecules. 2022 Oct 25;27(21):7226. doi: 10.3390/molecules27217226 (PMC9653668; doi:10.3390/molecules27217226)
Supplement: Supplementary file 1 [file molecules-27-07226-s001.zip › molecules-1971646-supplementary.pdf]

Supplementary Materials for the paper

Complex-forming properties of ceftazidime with Fe(III) ions in an aqueous solution

Marek Pająk, Magdalena Woźniczka and Jakub Fichna

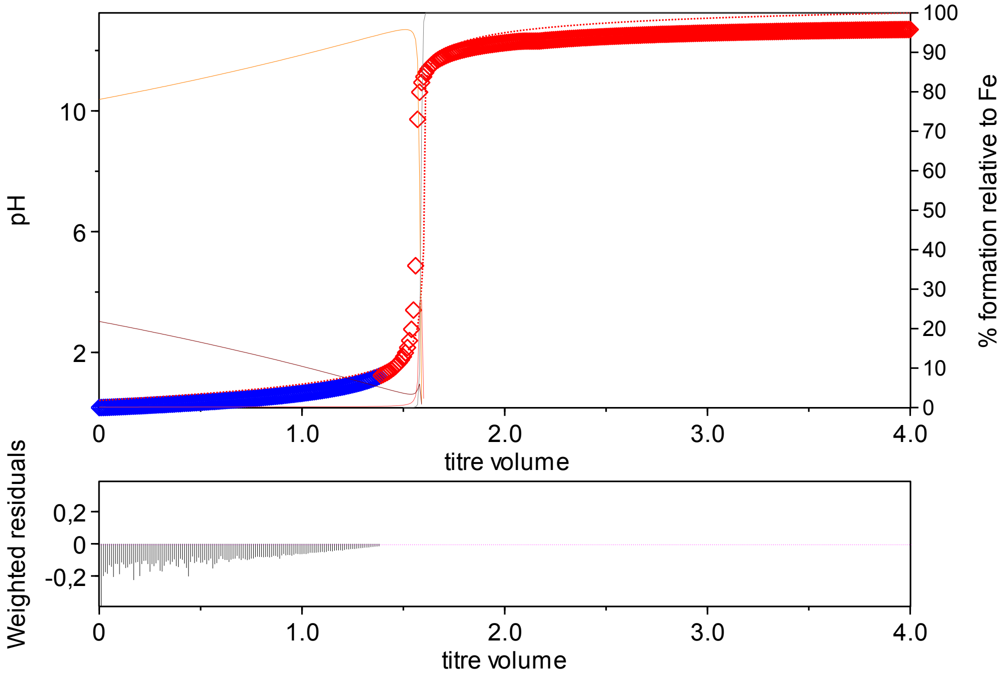

Figure S1. The pH-titration curves for the Fe(III) - ceftazidime system, at ligand : Fe(III) molar ratio 5:1, $C_{ceftazidime}$ = 3,6·10^{-3} M. ◊ the experimental points included in the calculations; ◊ the experimental points not included in the calculations; --- the theoretical values.
